# Clinical Outcomes after Revascularization in Patients with Chronic Limb-Threatening Ischemia

**DOI:** 10.3400/avd.ra.24-00135

**Published:** 2025-01-20

**Authors:** Akio Kodama

**Affiliations:** Department of Vascular Surgery, Aichi Medical University, Nagakute, Aichi, Japan

**Keywords:** chronic limb-threatening ischemia, revascularization, GLASS, quality of life

## Abstract

Chronic limb-threatening ischemia (CLTI) occurs in the advanced stage of peripheral artery disease and is associated with high risks of mortality and amputation. Universal management strategies are not always applicable, owing to population diversity, and the Western trials may not be applicable to Japanese patients, owing to differences in demographics and clinical profiles. This paper examines the outcomes of revascularization in Japanese CLTI patients and emphasizes the benefits of tailored management. Post-hoc analysis of the bypass versus angioplasty in severe ischemia of the leg (BASIL)-1 trial validated the use of the Global Limb Anatomic Staging System for predicting the outcomes of endovascular therapy (EVT) but not bypass surgery (BS). The SPINACH (surgical reconstruction versus peripheral intervention in patients with critical limb ischemia) registry revealed comparable 3-year amputation-free survival rates between patients who underwent EVT and those who underwent BS, with patient-specific factors such as limb status and general health influencing its success. Revascularization improved the quality of life, but benefits declined over time, especially in non-ambulatory and older patients on dialysis. Surgical reconstruction is better for preserving ambulation. Retrospective studies revealed pedal branch artery bypass as a viable option, functional independence as a predictor of survival, and zinc supplementation as promising for wound healing. Future research should focus on refining these strategies and exploring innovative approaches to overcome persistent challenges in CLTI care.

## Introduction

Chronic limb-threatening ischemia (CLTI) is the advanced stage of lower extremity artery disease, a condition that is becoming increasingly prevalent and contributes to rising healthcare costs worldwide. CLTI is associated with high rates of morbidity in addition to high risks of mortality, limb amputation and severe pain, not to mention a marked reduction in health-related quality of life (HR-QOL) for the affected individuals. Despite the involvement of various healthcare specialists in CLTI management, low public awareness and frequent delays in early diagnosis remain major barriers to effective treatment.^1)^

In general, CLTI affects individuals with diverse backgrounds, limb conditions, and anatomical characteristics, making it difficult to establish evidence thus far. In fact, the bypass versus angioplasty in severe ischemia of the leg (BASIL)-1 trial was the only RCT in which researchers compared the outcomes of endovascular therapy with those of bypass surgery for approximately two decades. However, the results of the BEST-CLI and BASIL-2 trials have been reported.^2)^ Moreover, the global vascular guidelines for the management of CLTI (GVG) were published in 2019.^1)^ Although these RCTs and guidelines had significant clinical impacts on the management of CLTI, the backgrounds of CLTI patients in Japan differ substantially from those in Western countries. Therefore, the results of these RCTs and some statements from the GVG cannot be directly applied to the treatment of CLTI patients in Japan. We must obtain evidence from studies involving the Japanese CLTI population.

Here, I provide an overview of studies in which I was involved and present the clinical outcomes of revascularization in CLTI patients.

## 1. Post hoc analysis of RCTs

### 1.1 Validation of the GLASS in the BASIL-1 trial^3)^

**Figure 1:**
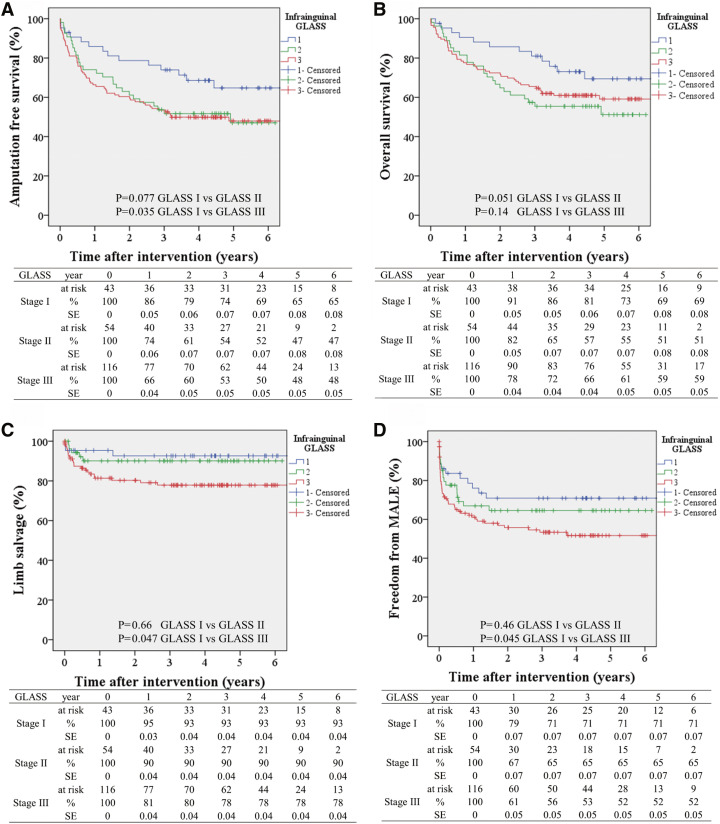
Fig. 1 Relationship between GLASS stage and long-term clinical outcomes following endovascular therapy for CLTI in the BASIL-1 trial for (**A**) amputation free survival, (**B**) overall survival, (**C**) limb salvage, and (**D**) freedom from MALE. BASIL: bypass versus angioplasty in severe ischemia of the leg; CLTI: chronic limb-threatening ischemia; GLASS: global limb anatomic staging system; MALE: major adverse limb event; SE: standard error.

The Global Limb Anatomic Staging System (GLASS) was proposed in the GVG as a new angiographic scoring system. However, the relationship between the GLASS stage and clinical outcomes following revascularization has not been previously studied. Therefore, we tried to apply the GLASS to the BASIL-1 cohort. This study analyzed the relationships between the GLASS stage and outcomes such as immediate technical failure (ITF), amputation-free survival (AFS), limb salvage (LS), overall survival (OS), and freedom from major adverse limb events (MALEs) in 377 patients who underwent either endovascular therapy (EVT, *n* = 213) or bypass surgery (BS, *n* = 164) as part of the BASIL-1 trial, conducted between 1999 and 2004, using preintervention angiograms (**Table 1**). The results revealed that there was no notable difference in the GLASS stage between the patient groups. However, a significant association was found between the ITF and GLASS stage in the EVT group (I 14%, II 15%, III 28%, p = 0.049). The GLASS stage was also significantly linked to AFS (hazard ratio [HR], 1.37; 95% CI 1.01–1.85; p = 0.042), LS (HR 1.96; 95% CI 1.12–3.43; p = 0.018), and freedom from MALEs (HR 1.49; 95% CI 1.04–1.87; p = 0.028) in patients who underwent EVT (**Fig. 1**). In contrast, no such relationships were observed in BS patients. The rate of freedom from MALEs was significantly lower after EVT than after BS in GLASS stage II (p = 0.038) and III (p = 0.001) patients. Furthermore, in the multivariable analysis, in the EVT cohort but not in the BS cohort, higher GLASS stages were significantly related to lower AFS and LS rates and higher MALE rates (**Table 2**).

**Table:** Table 1 Comparison of the baseline characteristics of patients undergoing bypass surgery and endovascular therapy for chronic limb-threatening ischemia (CLTI) in the bypass versus angioplasty in severe ischemia in the leg (BASIL)-1 trial

	Overall (n = 377)	EVT (n = 213)	BS (n = 164)	P value
Age (years, mean ± SD)	73 ± 9	74 ± 8	72 ± 10	0.11
Male	223 (59%)	113 (53%)	110 (67%)	<0.01
Non-ambulatory status	30 (8%)	16 (8%)	14 (9%)	0.71
Body mass index (median [IQR])	24 (22, 28)	25 (22, 28)	24 (22, 27)	0.80
*Comorbidities*				
Hypertension	239 (63%)	140 (66%)	99 (60%)	0.33
Diabetes	159 (42%)	91 (43%)	68 (41%)	0.83
*Smoking status*				
Current smoker	139 (37%)	68 (32%)	71 (43%)	0.08
Ex-smoker	159 (42%)	97 (46%)	62 (38%)	
Never smoker	79 (21%)	48 (23%)	31 (19%)	
Coronary artery disease	102 (27%)	63 (30%)	39 (24%)	0.24
Cerebrovascular disease	72 (19%)	37 (17%)	35 (21%)	0.36
Creatinine (µmol/L, median [IQR])	99 (84, 125)	98 (84, 124)	100 (84, 126)	0.39
*Preintervention medications*				
Antiplatelet agents	225 (60%)	118 (55%)	107 (65%)	0.06
Statins	130 (35%)	77 (36%)	53 (32%)	0.45
*Limb status*				
Tissue loss	286 (76%)	161 (76%)	125 (76%)	0.90
ABPI (mean ± SD)	0.49 ± 0.16	0.49 ± 0.16	0.49 ± 0.17	0.95
*Location of disease*				
FP/FP+IP/IP	218/97/12	159/49/5	109/49/6	0.21
*GLASS*				
FP grade (0/1/2/3/4)	13/43/61/65/195	5/23/37/42/106	8/20/24/23/89	0.35
IP grade (0/1/2/3/4)	254/41/36/14/32	149/23/18/8/15	105/18/18/6/17	0.68
Selected TAP (AT/PE/PT)	168/124/85	93/75/45	75/49/40	0.52
GLASS stage (I/II/III)	76/87/214	43/54/116	33/33/98	0.46

ABPI: ankle-brachial pressure index; GLASS: Global Anatomic Staging System; FP: femoro-popliteal; IP: infrapopliteal; TAP: target artery path; AT: anterior tibial artery; PE: peroneal artery; PT: posterior tibial artery

Data are presented as n (%), means ± standard deviations (SDs), or medians (interquartile ranges [IQRs]).

**Table:** Table 2 Relationship between baseline patient characteristics and long-term clinical outcomes following endovascular therapy and bypass surgery for CLTI in the BASIL-1 trial

*Outcome measure* (number of observed events)	Univariate analysis			Multivariate analysis		
	HR	95% CI	P value	HR	95% CI	P value
Endovascular therapy						
*Major amputation or all-cause death* (100)						
Male	1.66	1.11–2.48	0.014	1.96	1.20–3.21	0.008
Non ambulatory status	2.17	1.13–4.17	0.021	1.24	0.51–3.04	0.638
Body mass index (kg/m^2^, +1)	0.95	0.90–0.99	0.029	0.94	0.89–0.99	0.022
Coronary artery disease	1.70	1.13–2.54	0.010	1.39	0.85–2.28	0.186
Cerebrovascular disease	1.66	1.03–2.66	0.037	2.23	1.23–4.06	0.009
Tissue loss	3.44	1.84–6.44	<0.001	3.22	1.63–6.37	0.001
GLASS stage (stage, +1)	1.28	1.00–1.68	0.049	1.37	1.01–1.85	0.042
*Overall mortality* (83)						
Body mass index (kg/m^2^, +1)	0.93	0.89–0.99	0.013	0.94	0.89–0.99	0.020
Coronary artery disease	2.30	1.49–3.55	<0.001	2.75	1.58–4.80	<0.001
Serum creatinine (µmol/L, +1)	1.00	1.00–1.01	0.046	1.002	0.999–1.01	0.20
Statin use	0.60	0.37–0.97	0.038	0.57	0.31–1.06	0.074
Tissue loss	3.24	1.62–6.47	0.001	2.50	1.08–5.75	0.032
*Major amputation* (31)						
Diabetes	3.18	1.50–6.76	0.003	2.88	1.35–6.13	0.006
Tissue loss	5.60	1.34–23.50	0.018	5.10	1.21–21.45	0.026
GLASS (stage, +1)	1.90	1.09–3.33	0.025	1.96	1.12–3.43	0.018
*MALE* (81)						
Diabetes	2.22	1.41–3.50	0.001	2.11	1.34–3.27	0.001
Tissue loss	1.83	1.01–3.33	0.048	1.51	0.96–3.19	0.069
GLASS (stage, +1)	1.49	1.09–2.03	0.012	1.49	1.04–1.87	0.028
*Bypass surgery*						
*Major amputation or death* (71)						
Diabetes	1.92	1.20–3.06	0.006	1.77	1.10–2.85	0.019
Cerebrovascular disease	1.88	1.14–3.11	0.014	1.67	0.998–2.78	0.051
*Overall mortality* (57)						
Diabetes	1.67	0.99–2.80	0.054	1.60	0.95–2.72	0.080
Cerebrovascular disease	1.97	1.13–3.45	0.017	1.90	1.08–3.35	0.027
Statin use	0.59	0.32–1.10	0.096	0.54	0.29–1.004	0.052
*Major amputation* (28)						
Diabetes	2.08	0.98–4.40	0.055			
*MALE* (35)						
Diabetes	1.78	0.91–3.49	0.095			

Among patients with femoropopliteal (FP) disease (BS, n = 109; EVT, n = 159), the rates of freedom from MALEs were significantly higher following BS than after EVT (p < 0.001). The advantage of BS over EVT increased as the GLASS FP stage increased, especially in patients who had vein grafts. In conclusion, the GLASS stage was linked to outcomes after EVT but not after BS in the BASIL-1 cohort. While further validation in modern CLTI cohorts is needed, the GLASS is likely to be valuable in shared decision-making and for stratifying patients in future clinical trials.

This paper was selected as the “Editor’s Choice” in this volume of the European Journal of Vascular and Endovascular Surgery.

## 2. Prospective multicenter observational studies

Since the BASIL-1 trial was first published, most studies on CLTI have been single-center or retrospective, with no high-level evidence. During this time, there have been advancements in revascularization, particularly in endovascular devices and techniques, along with increases in the population of aging CLTI patients and the population of those with diabetes and renal disease. Therefore, a multicenter prospective study was conducted to clarify the clinical outcomes of EVT and bypass surgery in a real-world study of Japanese CLTI patients. The primary endpoint was the AFS rate 3 years after revascularization.^4)^

In the SPINACH (surgical reconstruction versus peripheral intervention in patients with critical limb ischemia) study, 548 Japanese CLTI patients were followed. This study revealed that the 3-year AFS rate did not differ between critical limb ischemia (CLI) patients who underwent surgical reconstruction and those who received endovascular therapy. The subsequent risk stratification analysis suggested that patients with severe CLI would be better suited for surgical reconstruction, whereas patients with a poor general condition would benefit more from endovascular therapy.^5)^ This study was important and informative. Since then, the results of many subanalyses of the findings of this study have been published^6^^–^^13)^ and are frequently cited in current Japanese guidelines.^14)^

### 2.1 HR-QOL over time after revascularization^12)^

One of the major goals of CLTI treatment is to maintain patient mobility and HR-QOL by relieving ischemic pain and healing ischemic wounds. It is essential to consider the procedure’s impact on HR-QOL when planning the appropriate treatment approach. An assessment of HR-QOL is also recommended in the GVG. However, HR-QOL has been assessed using instruments in only a few studies of CLTI patients. Most of these were retrospective, single-center studies that examined a small cohort of CLTI patients. Therefore, we analyzed the HR-QOL of CLTI patients over time after revascularization and identified the predictors of improved HR-QOL as well as survival in the SPINACH study cohort.

The outcome measures included disease-specific QOL assessed using the Vascular Quality of Life (VascuQOL) questionnaire and generic QOL was evaluated with the 36-item Short Form (SF) survey. These parameters were assessed at baseline and 3, 12, 24, and 36 months after treatment. The outcome measure in the current study was improved QOL, which was defined as being alive and an improvement in HR-QOL scores on the VascuQOL and SF-36 from baseline by more than the minimally important difference (MID). The MID was used to better express clinically important changes rather than only statistically significant changes. A general distinction may be made between distribution-based and anchor-based methods. Distribution-based methods determine the MID on the basis of the statistical characteristics of an obtained sample. Anchor-based methods determine the MID by relating questionnaire scores to other clinically relevant measures. However, no reliable anchor-based MID of the VascuQOL or SF36 for CLTI patients was available at the time, and the MID was determined to be half of one standard deviation for each baseline value using the distribution-based method.

**Figure 2:**
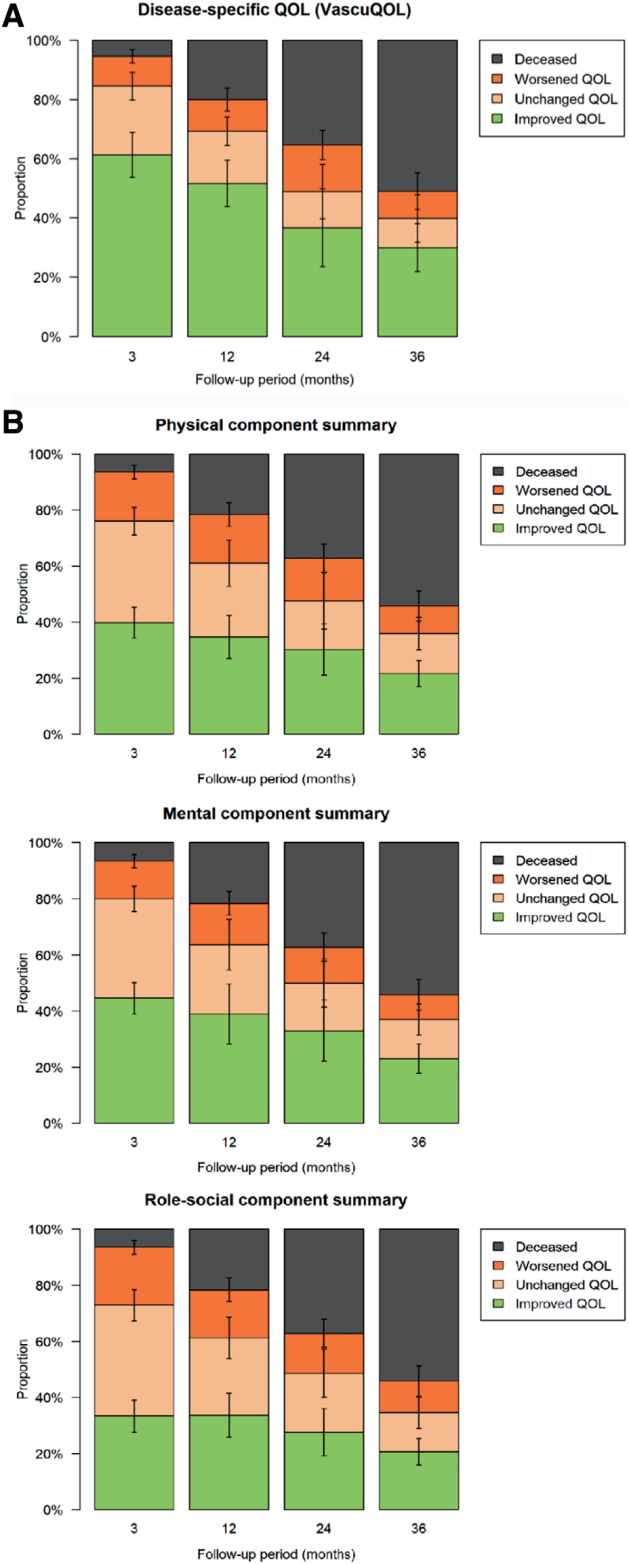
Fig. 2  (**A**) Change in HR-QOL according to the VascuQOL after revascularization in 415 patients with CLTI. Error bars indicate 95% confidence intervals. (**B**) Change in HR-QOL according to the Short Form 36 (SF-36) component summary for physical, mental, and role social components after revascularization in 415 patients with chronic limb threatening ischemia. Error bars indicate 95% confidence intervals. CLTI: chronic limb-threatening ischemia; HR-QOL: health-related quality of life; VascuQOL: vascular QOL.

The baseline characteristics of the study population are summarized in **Table 3**. The prevalence rates of diabetes mellitus and renal failure were 74% and 57%, respectively. For the SF-36 domains at baseline, patients in the current study perceived their HR-QOL to be much lower than that of the Japanese general population. In this study, 61% of patients showed an improvement in QOL on the basis of the VascuQOL (**Fig. 2A**), and approximately 40% showed improvement in the SF-36 score obtained 3 months after treatment (**Fig. 2B**). However, these proportions gradually decreased to 21%–31% over the 3-year period. Conversely, the percentage of deceased patients gradually increased throughout the follow-up period.

**Table:** Table 3 Baseline characteristics of the study population

	(n = 415)
Age (years)	73 ± 10
Male sex	284 (68%)
Non-ambulatory status	185 (45%)
Current smoking	72 (17%)
Body mass index (kg/m^2^)	22.1 ± 3.5
Diabetes mellitus	306 (74%)
Renal failure	238 (57%)
Heart failure	72 (17%)
WIfI stage	
Stage 2	67 (16%)
Stage 3	126 (30%)
Stage 4	222 (54%)
Surgical reconstruction	149 (36%)
Disease-specific QOL (VascuQOL)	2.4 ± 1.1
(missing data)	5 (1%)
Generic QOL (SF-36)	
Physical functioning	3 ± 17
(missing data)	25 (6%)
Role physical	18 ± 15
(missing data)	25 (6%)
Bodily pain	31 ± 11
(missing data)	25 (6%)
General health	35 ± 10
(missing data)	24 (6%)
Vitality	37 ± 12
(missing data)	25 (6%)
Social functioning	27 ± 15
(missing data)	26 (6%)
Role emotional	23 ± 17
(missing data)	26 (6%)
Mental health	36 ± 13
(missing data)	25 (6%)

Data are presented as n (%) or means ± standard deviations.

Multivariate analysis revealed that preoperative non-ambulatory status was negatively associated with improved QOL in both the 3-month VascuQOL and SF-36 mental component summary, whereas surgical reconstruction was positively associated with these outcomes. Advanced age and renal failure were inversely associated with improved QOL for both the SF-36 mental component summary and the VascuQOL between 1 and 3 years. In conclusion, revascularization improved QOL. However, a non-ambulatory status before the procedure was negatively associated with improved QOL 3 months after treatment, and advanced age and renal failure limited the benefits of revascularization 1–3 years later. The accumulation of QOL data will be crucial for assessing post-revascularization QOL. Preoperative evaluation, including estimated QOL, is important for shared decision-making and improving patient-centered outcomes following treatment of CLTI.

### 2.2 Ambulatory status over time after revascularization^13)^

The CLTI population is generally frail and limited in performing activities of daily living (ADL) and has limited lower limb functionality due to chronic ischemic pain and wounds. Furthermore, CLTI is strongly linked to high mid- and long-term mortality rates. Therefore, when making decisions about the most appropriate treatment, we should consider the patient's functional status and life expectancy. We examined ambulatory status over time after revascularization and the predictors of ambulation loss in the SPINACH study cohort. Ambulation loss indicated a wheelchair-bound, bedridden, or deceased status.

**Figure 3:**
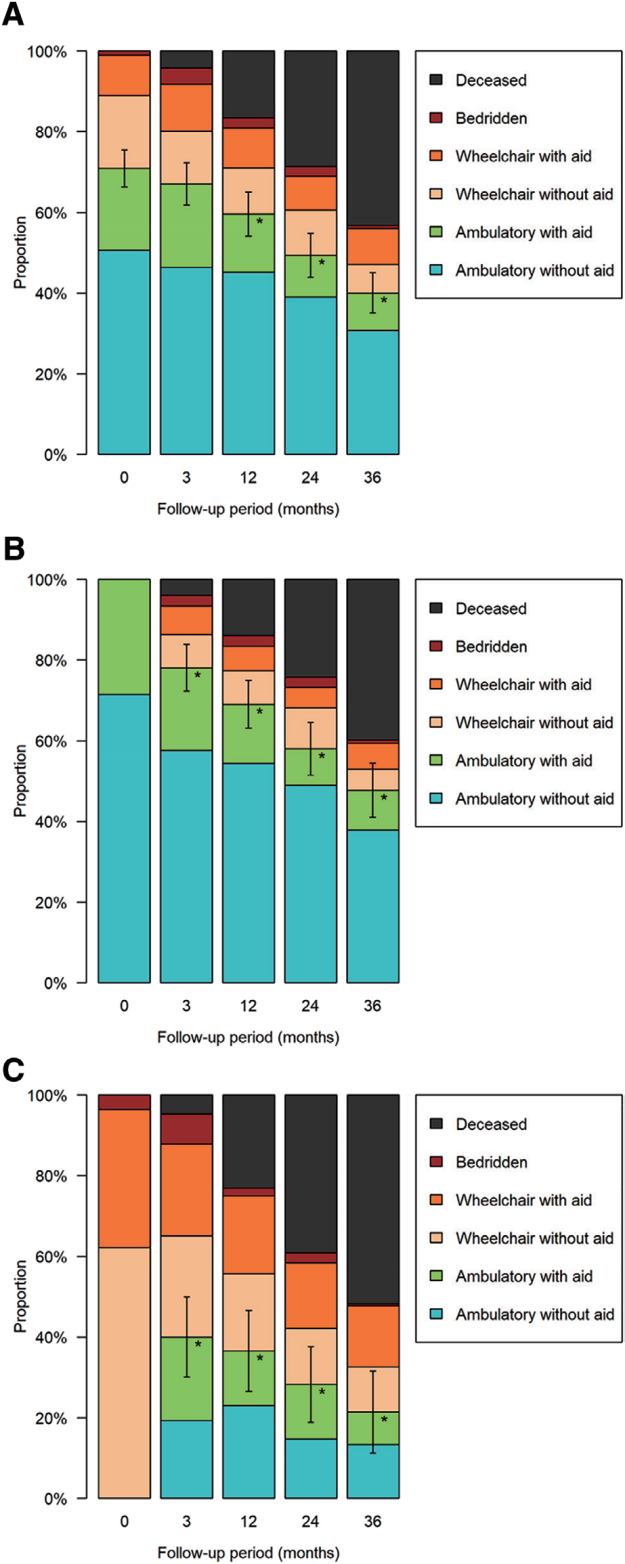
Fig. 3 Change in mobility status. Panels A–C illustrate the proportions of respective mobility statuses in the overall population (**A**), in patients who were ambulatory regardless of aid at baseline (**B**), and in those unable to ambulate (i.e., in a wheelchair regardless of aid or bedridden) at baseline (**C**). Error bars indicate 95% confidence intervals for the proportion of patients who were ambulatory regardless of aid, and asterisks represent P < 0.05 versus the baseline value regarding the proportion of patients who were ambulatory regardless of aid.

Among the 381 patients, 71% were ambulatory at baseline. This proportion gradually decreased, reaching 40% 36 months after intervention (**Fig. 3**). Approximately 20%–40% of non-ambulatory patients who underwent revascularization were able to ambulate postoperatively. Multivariate analysis revealed that factors such as age, impaired mobility before the onset of CLTI and at the time of revascularization, renal failure requiring dialysis, and WIfI clinical stage 4 disease were positively associated with the loss of ambulation at specific or all time points. In contrast, male sex and surgical reconstruction were negatively associated with ambulation loss at certain time points. In conclusion, mobility in the overall population gradually declined, whereas the number of deceased patients increased. Factors such as advanced age, impaired mobility before the onset of CLTI and at the time of revascularization, renal failure requiring dialysis, and WIfI stage 4 were associated with ambulation loss at nearly all time points following revascularization.

## 3. Retrospective multicenter observational studies

### 3.1 Clinical outcomes after inframalleolar bypass to the pedal branch artery

Although CLTI is generally accompanied by multilevel occlusive disease, infrapopliteal arterial disease is prevalent in patients with diabetes, who have small vessels, severe calcification and an extended disease course, making revascularization difficult. In inframalleolar bypass, the distal target artery is usually the dorsalis pedis artery or the common plantar artery. However, both arteries are sometimes occluded with patent branches (tarsal and plantar arteries) that serve as recipient vessels for bypass grafts.

In this study, we evaluated recent long-term clinical outcomes, including wound healing and graft patency, in patients following pedal branch artery (PBA) bypass and compared the results of inframalleolar (IM) bypass to the pedal artery (PA) with those of bypass to the PBA.

We analyzed the clinical data of 208 CLTI patients. Among the 208 patients, 174 (74%) underwent PA bypass, whereas 34 (16%) underwent PBA bypass. In the PBA group, 32 patients (94%) had P2 IM disease according to the GLASS. In the PBA group, 14 distal anastomoses were observed in the medial plantar artery, 13 were found in the lateral plantar artery, 4 were observed in the medial tarsal artery, and 3 were found in the lateral tarsal artery.

**Figure 4:**
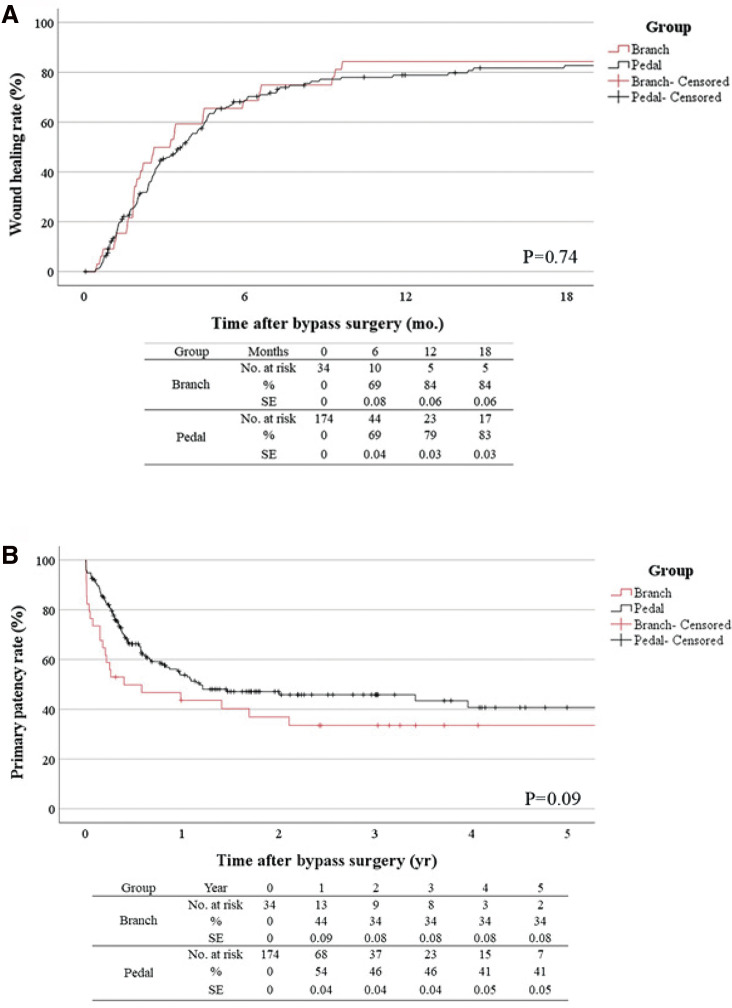
Fig. 4 (**A**) Comparison of the wound healing rate between the PBA bypass group and the PA bypass group. (**B**) Comparison of primary patency between the PBA bypass group and PA bypass group. PA: pedal artery; PBA: pedal branch artery; SE: standard error

Early (30-day) graft failure was more common in the PBA group. The cumulative wound healing rate was similar in both groups (**Fig. 4A**, P = 0.74). The rates of primary patency in the PA group and the PBA group were 54% and 44%, respectively, at 1 year and 46% and 34%, respectively, at 3 years (**Fig. 4B**, P = 0.09). In the multivariate analysis, the GLASS IM grade (HR, 0.73; 95% confidence interval [CI], 0.58–0.93: P = 0.006), WIfI wound grade (HR, 0.67; 95% CI, 0.51–0.89: P <0.01), and WIfI foot infection grade (HR, 0.79; 95% CI, 0.65–0.96: P = 0.02) were associated with wound healing. The GLASS IM grade was also associated with loss of primary patency (HR, 1.65; 95% CI, 1.19–2.29: P = 0.003), MALEs (HR, 2.53; 95% CI, 1.63–3.93: <0.001), and major amputation (HR, 3.27; 95% CI, 1.66–6.47: P = 0.001). However, the difference between the two groups (i.e., the PBA and PA groups) was not associated with these midterm clinical outcomes.

Recently, percutaneous deep venous arterialization (pDVA) has been proposed as an alternative to major amputation for “no-option” CLTI patients, particularly those with diabetes or who are on hemodialysis. However, the definition of “no-option” varies among clinicians and can include the absence of distal runoff or occluded pedal vessels. According to the GVGs, the absence of a target artery crossing the ankle or a suitable pedal/plantar artery (e.g., the GLASS P2 modifier) indicates a no-option disease pattern. In this study, 94% of the PBA group had the GLASS P2 modifier. Thus, some physicians may not consider these cases “no-option” because they could perform PBA bypass, and others may consider these cases “no-option” and perform pDVA. Some comparative studies between PBA bypass and pDVA with a patent PBA are warranted in the future.

### 3.2 Relationship between clinical outcomes and cognitive function^15)^

Several studies have revealed an association between motor ADL evaluated by the Barthel Index (BI) and prognosis in patients with CLTI, which indicates the importance of such activities in these patients.^16)^ However, the relationship between cognitive function and clinical outcomes in patients who undergo lower limb revascularization remains unclear. Therefore, we examined the effects of preoperative motor and cognitive function on long-term outcomes in patients with CLTI after distal bypass surgery via the functional independence measure (FIM). The FIM is an 18-item global measure of independence in performing ADLs that is subdivided into 2 subscales: FIM-motor (13 items covering self-care, sphincters, transfers, and locomotion) and FIM-cognitive (5 items covering communication and social cognition). Each item is scored from 1 (total dependence) to 7 (full independence).^17)^

A retrospective review of the clinical data of patients who underwent distal bypass surgery for CLTI after ADL evaluation at 3 centers in Japan between January 2013 and December 2019 was performed. On the basis of the standards of the Japanese Ministry of Health, Labor and Welfare, FIM-motor high and low cases were defined as those with FIM-motor scores >75 and ≤75 points, and FIM-cognitive high and low cases were defined as those with FIM-cognitive scores >24 and ≤24 points.

A total of 226 distal bypass surgeries were conducted on 185 patients (169 males; median age, 76 years). Among them, 70% had diabetes mellitus and 40% had end-stage renal disease requiring hemodialysis.

**Figure 5:**
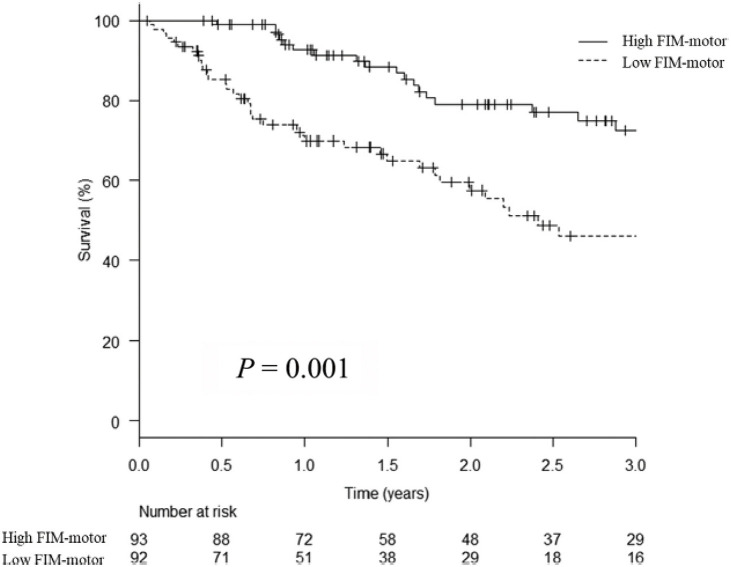
Fig. 5 Kaplan‒Meier life-table analysis of survival of CLTI patients after distal bypass surgery in low and high FIM-cognitive cases. CLTI: chronic limb-threatening ischemia; FIM: functional independence measure

The patients were categorized into high (n = 93, 50%) and low (n = 92, 50%) FIM-motor groups, and high (n = 157, 85%) and low (n = 28, 15%) FIM-cognitive groups. No significant associations were found between FIM-motor or FIM-cognitive levels and limb salvage, freedom from MALEs, readmission, or wound healing rates. However, survival rates at 1 and 3 years were significantly lower in the low FIM-motor group (1-year: 93% vs. 70%; 3-year: 73% vs. 46%, P = 0.0011) and the low FIM-cognitive group (1-year: 87% vs. 50%; 3-year: 63% vs. 45%, P < 0.001). Similarly, the 1- and 3-year AFS rates were significantly lower in the low FIM-motor group (1-year: 92% vs. 67%; 3-year: 69% vs. 44%, P < 0.001) and the low FIM-cognitive group (1-year: 85% vs. 49%; 3-year: 59% vs. 44%, P < 0.001; **Fig. 5**). In the multivariate analysis, hemodialysis (HR = 2.17; 95% CI, 1.23–3.83; P = 0.0078), low FIM-cognitive score (HR = 3.45; 95% CI, 1.78–6.71; P < 0.001), and ejection fraction (HR = 0.98; 95% CI, 0.95–0.99; P = 0.019) were identified as independent risk factors for survival.

In conclusion, FIM-motor and FIM-cognitive scores are predictors of long-term survival and AFS of CLTI patients following distal bypass surgery. These findings indicate that assessing motor and cognitive ADL status via FIM prior to distal bypass surgery may be valuable for patients with CLTI.

### 3.3 Impact of zinc level and zinc supplementation on wound healing in CLTI patients

Zinc (Zn) is an essential micronutrient involved in numerous biological functions; it binds to more than 300 enzymes and 2000 transcription factors and is key to wound healing. Recent studies have linked Zn deficiency with cardiovascular diseases, especially atherosclerosis. Despite CLTI being a severe form of atherosclerosis, the association between Zn deficiency and CLTI remains underreported. Therefore, we examined the association between Zn deficiency and clinical outcomes in CLTI patients undergoing bypass surgery, and, if an association was found, to determine whether oral Zn supplementation could improve these outcomes.

In this study, the clinical data of 111 consecutive patients who underwent infrainguinal bypass from 2012 to 2020 were reviewed. Patients with Zn deficiency (serum Zn level <60 μg/dL) received oral Zn supplementation and maintained a normal level until wound healing (WH). The aim of this study was to explore (1) the effect of Zn deficiency and (2) the effect of Zn supplementation in Zn-deficient patients on the clinical outcomes of this cohort.

**Figure 6:**
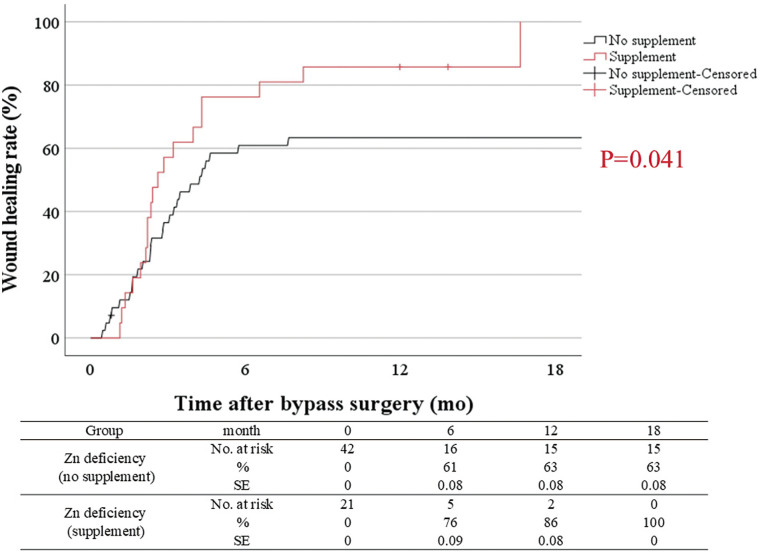
Fig. 6 Wound healing between the zinc (Zn) supplementation for deficiency group (preoperative serum Zn level <60 μg/dL and supplementation after bypass), and the no Zn supplementation despite deficiency group (preoperative serum Zn level <60 μg/dL but no Zn supplementation) regarding long-term clinical outcomes in patients who underwent bypass surgery for CLTI. CLTI: chronic limb-threatening ischemia

In sum, 48 patients were Zn sufficient, 21 patients received Zn supplementation for Zn deficiency, and 42 patients did not receive Zn supplementation for Zn deficiency. (1) Zn deficiency was associated with WH (HR, 0.47; 95% CI, 0.29–0.78: P = 0.003), MALEs (HR, 2.53; 95% CI, 1.26–5.09: P = 0.009), and major amputation or death (HR, 3.17; 95% CI, 1.51–6.63: P = 0.002). (2) The cumulative WH rate in patients with lower Zn levels and those who received Zn supplementation was significantly higher than that in those who did not receive Zn supplementation (P = 0.041, **Fig. 6**). On multivariate analysis, Zn supplementation was positively related to WH (HR, 2.30; 95% CI, 1.21–4.34: P = 0.011). This result was confirmed via propensity score matching (HR, 2.24; 95% CI, 1.02–4.87: P = 0.043).

This study revealed that Zn could influence some clinical outcomes in CLTI patients undergoing bypass surgery. Prospective multicenter randomized controlled trials are needed to validate the findings of this study.

## 4. Retrospective single-center observational studies

### 4.1 Clinical outcomes after bypass surgery in dialysis-dependent patients with CLI^18)^

Although more than half of patients with CLI in Japan are dialysis patients, in general, the combination of CLI and dialysis-dependent end-stage renal disease (ESRD) has been associated with low medium- and long-term survival rates.^19)^ However, few small- to medium-sized studies have addressed infrainguinal bypass grafting (IBG) in patients with ESRD. Moreover, little information on the predictors of short- and medium-term outcomes after IBG in hemodialysis patients is available. Therefore, we aimed to evaluate the short- and medium-term outcomes of infrainguinal bypass grafting in ESRD patients at a single institution in Japan and to identify preoperative factors predictive of 30-day mortality, AFS, freedom from MALEs, limb salvage, and overall survival.

The clinical data of consecutive patients with critically ischemic limbs and peripheral arterial disease who had undergone de novo infrainguinal revascularization at the authors’ hospital from April 2007 to March 2011 were analyzed retrospectively. During the study period, IBG was performed in 162 patients (198 limbs), EVT in 221 patients (260 limbs), and primary amputation in 21 patients (21 limbs). Among these patients, 186 (229 limbs) with ESRD presented with CLI. The 30-day mortality rate was 9%, and the only positive predictor of 30-day mortality was an ejection fraction (EF) <40%. The cumulative mortality rate was 49.6% at 2 years after the intervention. The rate of freedom from MALEs was 77.4%, and the rate of freedom from amputation was 85.0%. Additionally, a multi-regression analysis revealed that an ejection fraction <40%, a serum albumin level <3.0 g/dL, and non-ambulatory status were independent predictors of a poor outcome (**Table 4**).

**Table:** Table 4 Midterm outcomes and predictors based on multivariate analysis

Clinical outcomes	Predictors	HR	95% CI	*p*
Amputation free survival	Non-ambulatory status	3.04	1.59–5.82	<0.01
Freedom from MALE	Non-ambulatory status	4.98	1.91–12.96	<0.01
Limb salvage	Non-ambulatory status	5.18	1.47–18.30	0.01
Survival	Non-ambulatory status	2.35	1.19–4.64	0.01
	Albumin <3.0 g/dL	2.26	1.12–4.58	0.02
	Ejection fraction <40%	2.24	1.05–4.79	0.04

HR: hazard ratio; CI: confidence interval; MALEs: major adverse limb events

### 4.2 Relationship between preoperative frailty and mortality in patients with CLI^16)^

Owing to the aging of society, frailty has become a focus of increasing attention in recent years. Some studies have validated multiple assessments to identify frail patients on the basis of the recognition of numerous physiological domains (e.g., comorbidity, physical function, nutrition, cognition, geriatric syndrome, and social vulnerability). Nevertheless, there is no single gold standard measure of frailty.^20)^

Patients with CLI often present with significant comorbidities and an impaired functional status. This is primarily attributable to their limited ability to engage in adequate physical activity, a consequence of ischemic symptoms such as pain at rest, tissue loss, gangrene, and cardiopulmonary dysfunction. Consequently, their physiological reserves are frequently diminished, and a substantial proportion of CLI patients also exhibit characteristics of frailty. The aim of this study was to evaluate the utility of the BI as a tool for risk stratification following bypass surgery. The BI is a 10-item scale that assesses a patient’s ability to feed, groom, bathe, use a toilet, dress, walk, transfer, and climb stairs themselves, as well as fecal incontinence and urinary incontinence.^21)^ The BI is calculated by adding 5, 10, or 15 points for the presence of each variable (final score 0–100 points).

In this study, the clinical data of 107 CLI patients who underwent infrainguinal bypass surgery at a single institution were analyzed. Consequently, atrial fibrillation and the ejection fraction, body mass index, and BI were associated with mortality. Overall survival rates at 1 and 3 years after IB surgery were significantly lower in the group with a lower BI (69% and 32%, respectively) than in the group with a higher BI (96% and 77%, respectively; P < 0.001). Even after matching for differences in patient characteristics, the group with a lower BI had a significantly lower rate of survival than the group with a higher BI did (P = 0.02).

## Conclusion

This review focuses on the validity of the GLASS classification in the BASIL-1 trial, one of the few randomized controlled trials comparing revascularization strategies for CLTI, and the ability of participants in the SPINACH study, a multicenter prospective study conducted in Japan with significant impact, to perform ADLs as well as their HR-QOL. Furthermore, given that clinical outcomes in CLTI patients are not solely determined by the method of revascularization, additional analyses focused on frailty, cognitive function, and trace elements were conducted.

These findings may contribute to the treatment of CLTI, which requires a diverse and multifaceted approach. More research is warranted to confirm these findings in the future.

## Declarations

### Disclosure statement

The authors declare that they have no conflicts of interest.

### Author contributions

Study conception: AK

Data collection: AK

Analysis: AK

Investigation: AK

Manuscript preparation: AK

Funding acquisition: AK

Critical review and revision: AK

Final approval of the article: AK

Accountability for all aspects of the work: AK.
